# Polyoma Virus-Induced Osteosarcomas in Inbred Strains of Mice: Host Determinants of Metastasis

**DOI:** 10.1371/journal.ppat.1000733

**Published:** 2010-01-22

**Authors:** Palanivel Velupillai, Chang Kyoo Sung, Yu Tian, Jean Dahl, John Carroll, Roderick Bronson, Thomas Benjamin

**Affiliations:** Department of Pathology, Harvard Medical School, Boston, Massachusetts, United States of America; University of Wisconsin-Madison, United States of America

## Abstract

The mouse polyoma virus induces a broad array of solid tumors in mice of many inbred strains. In most strains tumors grow rapidly but fail to metastasize. An exception has been found in the Czech-II/Ei mouse in which bone tumors metastasize regularly to the lung. These tumors resemble human osteosarcoma in their propensity for pulmonary metastasis. Cell lines established from these metastatic tumors have been compared with ones from non-metastatic osteosarcomas arising in C3H/BiDa mice. Osteopontin, a chemokine implicated in migration and metastasis, is known to be transcriptionally induced by the viral middle T antigen. Czech-II/Ei and C3H/BiDa tumor cells expressed middle T and secreted osteopontin at comparable levels as the major chemoattractant. The tumor cell lines migrated equally well in response to recombinant osteopontin as the sole attractant. An important difference emerged in assays for invasion in which tumor cells from Czech-II/Ei mice were able to invade across an extracellular matrix barrier while those from C3H/BiDa mice were unable to invade. Invasive behavior was linked to elevated levels of the metalloproteinase MMP-2 and of the transcription factor NFAT. Inhibition of either MMP-2 or NFAT inhibited invasion by Czech-II/Ei osteosarcoma cells. The metastatic phenotype is dominant in F1 mice. Osteosarcoma cell lines from F1 mice expressed intermediate levels of MMP-2 and NFAT and were invasive. Osteosarcomas in Czech-II/Ei mice retain functional p53. This virus-host model of metastasis differs from engineered models targeting p53 or pRb and provides a system for investigating the genetic and molecular basis of bone tumor metastasis in the absence of p53 loss.

## Author Summary

The oncogenic mouse polyoma virus and its mutants have previously been used to investigate viral determinants of tumor induction using a standard inbred mouse strain as a common host. Here we use wild type virus to investigate the role of the host genetic background, focusing on two host strains that differ with respect to bone tumor metastasis. Comparing osteosarcoma cell lines from these mice, we have identified a molecular pathway that underlies invasive behavior *in vitro* and correlates with metastasis *in vivo*. The pathway involves secretion of the metalloproteinase MMP-2 under partial control of NFAT as a transcriptional regulator. This virus-host system reflects an important feature of human osteosarcoma with respect to pulmonary metastasis. Based on naturally occurring differences among inbred mice, the model differs from genetically engineered models targeting p53 or pRb as known risk factors in the human disease. Here, metastatic osteosarcomas retain functional p53. As noted by others, the frequency of p53 loss in patients with localized versus metastatic disease is the same, suggesting that events beyond p53 loss are important in metastasis. While the downstream effectors of metastasis in the genetically engineered models remain unknown, evidence presented here implicates upregulation of an NFAT → MMP-2 pathway in the development of metastatic osteosarcoma.

## Introduction

Invasion and metastasis are major factors underlying cancer morbidity and mortality [1]. Mouse models have been developed for many types of human cancer though not all present the same biological behavior as human tumors with respect to invasion and metastasis. Some models are of spontaneous origin while most are based on genetically engineered animals. Transgenic mice, mice with germline or conditional knockouts, targeted mutations [2], or ones emerging from screens following germline mutagenesis [3] have all been used to establish experimental models of specific types of cancer. Mice genetically engineered to develop cancer are typically derived from relatively few inbred strains thus reflecting a limited range of effects imposed by the host genetic background. Crosses of genetically engineered mice to other strains have been useful for identifying tumor modifiers [4],[5].

The mouse polyoma virus (Py) is a small DNA virus that rapidly induces a variety of solid tumors in its natural host under laboratory conditions [6]. The virus establishes a disseminated infection using specific gangliosides as broadly expressed cell receptors [7]. Features of the major viral capsid protein VP1 are important in receptor discrimination and polymorphisms in VP1 are major determinants of pathogenicity [8],[9]. The replication and transforming functions of the virus reside in the T (tumor) antigens. These have been extensively characterized using cell culture systems. Expression of the T antigens leads to alterations in the regulation of growth and apoptotic pathways of the cell. These alterations involve both protooncogene activation and tumor suppressor gene inactivation. Well characterized wild type and mutant virus strains have been used to assess the roles of specific viral determinants in tumorigenesis using a standard inbred mouse strain as the common host [10]–[13].

The virus can be introduced into any of a large number of inbred strains of mice to investigate effects of the host genetic background on various aspects of tumor development. Inbred mice vary greatly with respect to overall tumor frequency, spectrum of tumor types, and individual tumor behavior [6]. Py-induced tumors typically remain at the site of origin enclosed within a basement membrane. Susceptible hosts develop multiple tumors and may carry a cumulative tumor load in excess of 25% of total body weight while showing no evidence of metastasis [14]. The middle T antigen, the major viral transforming protein, activates multiple signal transduction pathways essential for tumor induction [15]. Transgenic mice expressing middle T under control of the mouse mammary tumor virus regulatory region develop mammary tumors which metastasize to the lung [16]. In this transgenic mouse system, secretion of osteopontin (OPN) was found to be necessary but not sufficient for metastasis [17].

In a survey of over thirty inbred mouse strains inoculated with Py [6], the Czech II/Ei mouse (CZ) emerged with unique and interesting properties. In addition to a typical array of polyoma tumors [18], CZ mice occasionally developed small nodules on the lung, normally not a site of tumor induction by the virus. These nodules proved to be metastatic lesions derived from osteosarcomas. The metastatic behavior of bone tumors in the Py-infected CZ mouse matches important clinical and pathological features of human osteosarcoma with respect to lung metastases [19]. C3H/BiDa mice (C3), a standard susceptible strain [20], develop osteosarcomas that fail to metastasize. Here we begin to explore the CZ mouse as a model of metastatic osteosarcoma using the C3 mouse as a control. A molecular pathway has been identified that correlates with properties of invasion *in vitro* and metastasis *in vivo*.

## Results/Discussion

### Bone tumor metastasis to the lung is determined by the host genetic background

**Figure 1:**
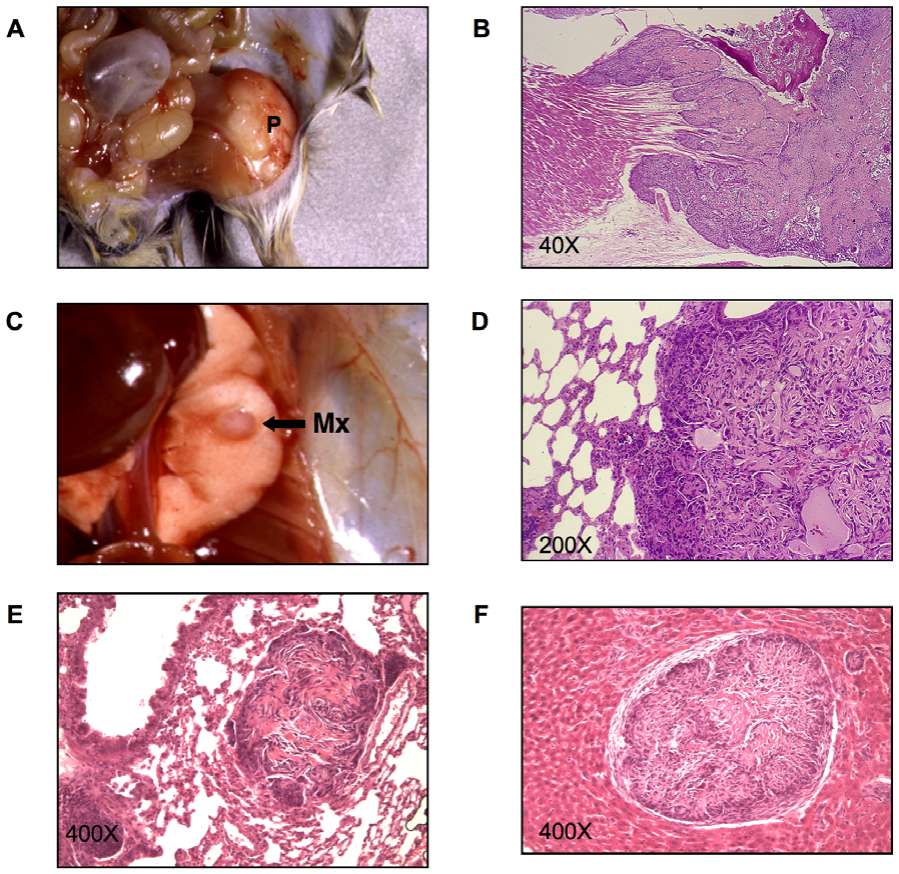
Primary and metastatic bone tumors from CZ mice. A) A primary bone tumor (P) on the head of the femur of a CZ mouse. B) H&E stained section of the same tumor showing invasion of adjacent muscle and destruction of bone by invading tumor cells. C) Metastatic nodule (Mx) on the surface of the lung of the same animal. D) H&E stained section of the lung metastasis from the same animal showing osteiod. E) Occult metastatic lesion with osteoid in the lung of a different CZ animal. F) H&E stained section of a liver metastasis showing osteoid in a CZ animal inoculated with osteosarcoma cells.

Osteosarcomas have previously been noted in Py-infected mice of several inbred strains but with no evidence of metastasis. In CZ mice, however, these tumors have been found to metastasize regularly to the lung. Osteosarcomas develop most frequently on the femur, skull, ribs or other long bones, and occasionally on the spine and sternum. A typical case in a CZ mouse is shown in Figure 1. The primary tumor arose on the head of the femur (panel A). This tumor showed evidence of invasion of adjacent muscle and destruction of bone (panel B). A metastatic lesion was evident as a smooth shiny nodule on the surface of the lung (panel C). This nodule showed abundant tumor cells mixed with deposits of osteoid (panel D). Lung metastases were seen in a high percentage of CZ mice by routine step-sectioning and histological examination. These lesions consistently showed production of osteoid (panel E). Metastases were noted rarely in the liver. To confirm that the liver lesions in Py-infected CZ mice also derived from osteosarcomas, cells from a primary bone tumor were inoculated subcutaneously into an uninfected CZ mouse. This animal showed growth of tumor cells in the liver with deposits of osteoid (panel F). The presence of osteoid in metastastic lesions in CZ mice confirms their origins from osteosarcomas and not from other tumor types arising in the same animals. Further support derives from the fact that no lung metastases were noted in CZ mice that failed to develop primary osteosarcoma while developing other kinds of tumors.

Osteosarcomas in C3 mice resembled those in CZ mice grossly and histologically. However, these tumors showed no evidence of lung metastasis using the same search criteria as for CZ mice. A direct comparison of the frequencies of primary and metastatic osteosarcoma in CZ and C3 mice highlights important differences between these strains (Table 1). A high proportion of CZ mice developed osteosarcomas and 90% of these were found to have metastasized to the lung. The actual frequency of lung metastasis in CZ mice may approach 100% given the likelihood that not all lesions will be found in the step sectioning process. Osteosarcomas in C3 mice were found at a lower frequency due in part to higher frequencies of other tumor types that develop early and require the animals to be sacrificed. Nevertheless, of 18 cases of osteosarcoma in C3 mice, none were found to have metastasized to the lung. The sharp difference in frequency of metastasis between hosts is not due to differences in the time allowed for tumor development. The average ‘time to necropsy’ was 190 days for CZ and 188 for C3, with ranges of 104–514 days for CZ and 70 – 655 for C3.

**Table 1:** Host control of metastasis in CZ, C3 and [CZ x C3] F1 mice.

Mouse strain	No. of animals	Osteosarcoma	Lung Mx (%)
CZ	38	33	30 (91)
C3	106	18	0
[CZ X C3] F1	16	12	11 (92)

[CZ x C3] F1 mice were infected and followed for the development of osteosarcoma and then screened for lung metastases. These mice were found to resemble their CZ parent with respect to the high frequency and metastatic behavior of osteosarcomas (Table 1). The dominance of the metastatic phenotype suggests that CZ mice express a heritable factor(s) which promotes bone tumor metastasis and which may be lacking or insufficient in C3 mice.

### CZ and C3 bone tumor cells secrete and migrate equally well in response to osteopontin

**Figure 2:**
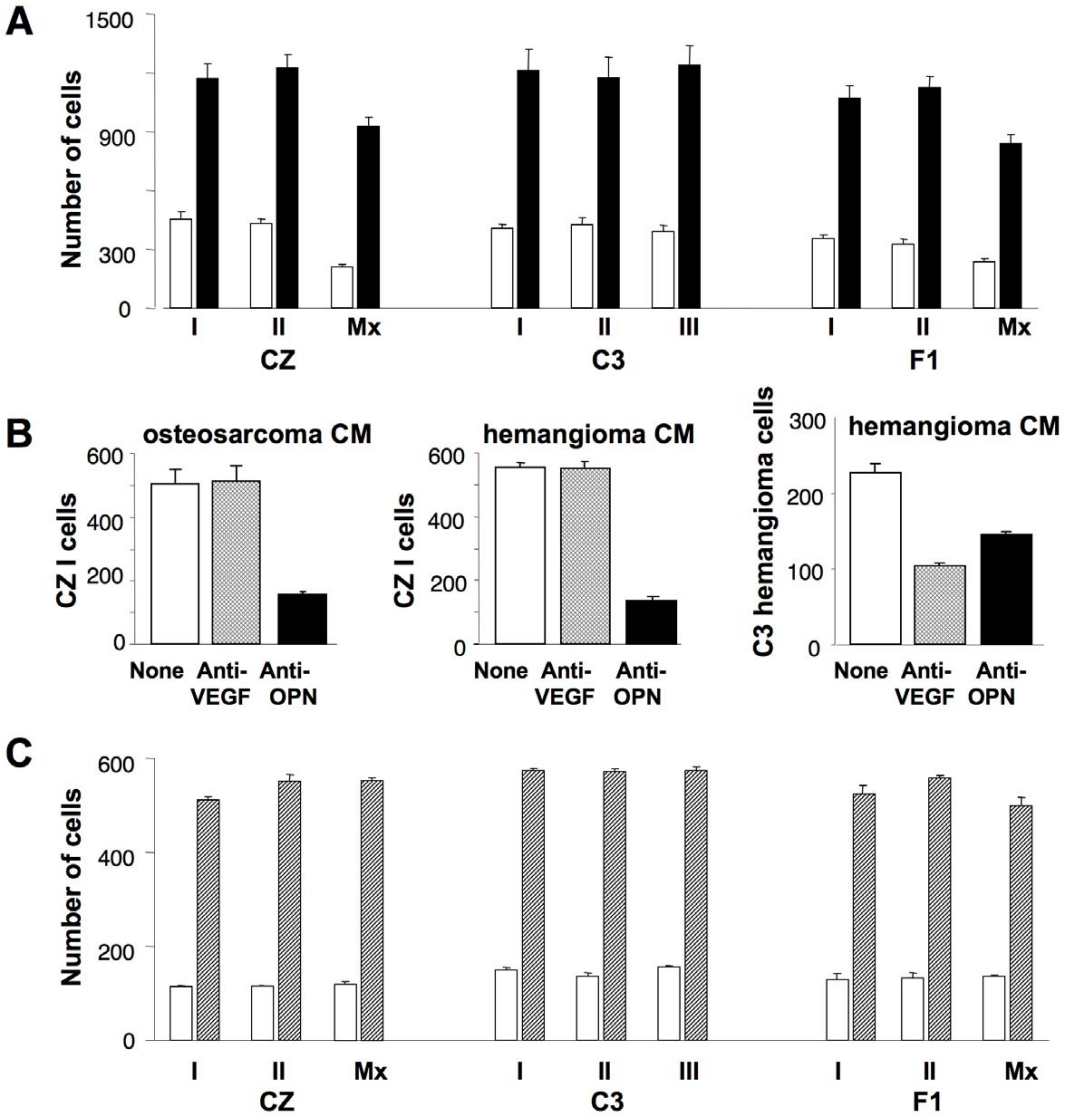
Osteopontin is the major chemoattractant inducing bone tumor cell migration. A) Bone tumor cells (3×10^5^) were used in a Boyden chamber assay using conditioned medium from C3 bone tumor cells as chemoattractant. Vertical bars represent mean number of cells migrating ± S.D. Solid bars show migration using conditioned medium. Empty bars show migration using serum-free medium as a control. B) Tumor cell migration was assayed as in (A) using conditioned medium (CM) that was untreated (empty bars), pre-treated with anti-VEGF (hatched bars) or anti-OPN (solid bars). Left panel – CZ I cells with osteosarcoma CM; Middle panel – CZ I cells with hemangioma CM; Right panel – hemangioma cells with hemangioma CM. C) Tumor cell migration in response to recombinant OPN in serum-free medium (hatched bars) or serum-free medium alone (empty bars).

To investigate the molecular basis of the metastatic phenotype, cell lines were established from primary osteosarcomas, two from CZ, three from C3 and two from F1 mice. Two additional lines were derived from lung metastatic lesions, one from a CZ and one from an F1 animal. It should be noted that no osteosarcomas were found in uninfected mice of either strain and expression of the Py T antigens was confirmed in each of the cell lines from infected mice (data not shown). The abilities of the tumor cells to migrate in response to a source of chemoattractant were tested in a transwell assay using a Boyden chamber with polycarbonate filter (8 µm pore size). Conditioned medium from a C3 osteosarcoma line was used as the attractant and serum-free medium as a control. Migration was stimulated 3–4 fold over a one hour period in response to the conditioned medium. No significant differences were noted among the lines (Figure 2A).

To identify the chemoattractant(s), the conditioned medium was first treated with antibodies to OPN or to vascular endothelial growth factor (VEGF). These factors, well recognized for their chemoattractant as well as mitogenic activities, play roles in normal bone development as well as tumor cell invasion and metastasis. OPN is of particular interest because expression of middle T has been shown to lead to transcriptional activation of OPN via two distinct pathways [21]. Furthermore, in an *in vitro* ‘wound healing’ assay, blocking induction of OPN with anti-sense RNA inhibited cell migration without interfering with transformed cell growth [21]. Pretreatment of conditioned medium with anti-OPN resulted in significant reduction in the migration of CZ-I cells while anti-VEGF had no effect (Figure 2B, left panel). Results were the same using the other lines as responding cells (data not shown). To determine whether the pathways from middle T to OPN operated equally in the two host backgrounds, levels of OPN were compared in conditioned media prepared from CZ and C3 bone tumor lines. Levels were found to be high and comparable between the two groups: 1975±262 pg/ml for the two CZ lines and 1592±38 pg/ml for the three C3 lines. Similarly, levels of VEGF, though much lower, were comparable: 153±12 pg/ml for CZ and 225±13 pg/ml for C3.

Additional experiments were carried out to test the specificity of migration in relation to these factors and also with respect to tumor type. Conditioned medium from a Py-induced hemangioma in a C3 mouse contained a higher level of VEGF (792±38 pg/ml) and a similar level of OPN (1622±20 pg/ml) compared to osteosarcoma conditioned media. Migration of CZ cells was again inhibited only by anti-OPN and not by anti-VEGF (Figure 2B, middle panel). In contrast, migration of the hemangioma cells in response to its own conditioned medium was inhibited by anti-VEGF as well as anti-OPN (Figure 2B, right panel). These results confirm OPN as the major attractant for osteosarcoma cells. They also demonstrate specificity in migratory behavior based on tumor type as well as attractant.

The osteosarcoma lines were compared for their abilities to migrate in response to recombinant OPN added to serum-free medium as the sole attractant. All nine lines responded well with no significant differences among the lines (Figure 2C). We conclude that the difference in metastatic behavior between CZ and C3 osteosarcomas is not due to a difference either in production of OPN or in ability to migrate in response to this factor. This conclusion is consistent with studies of mammary tumor metastasis in Py middle T transgenic mice which indicated that factor(s) in addition to OPN are required for metastasis [17].

### CZ but not C3 bone tumor cells show properties of invasion

**Figure 3:**
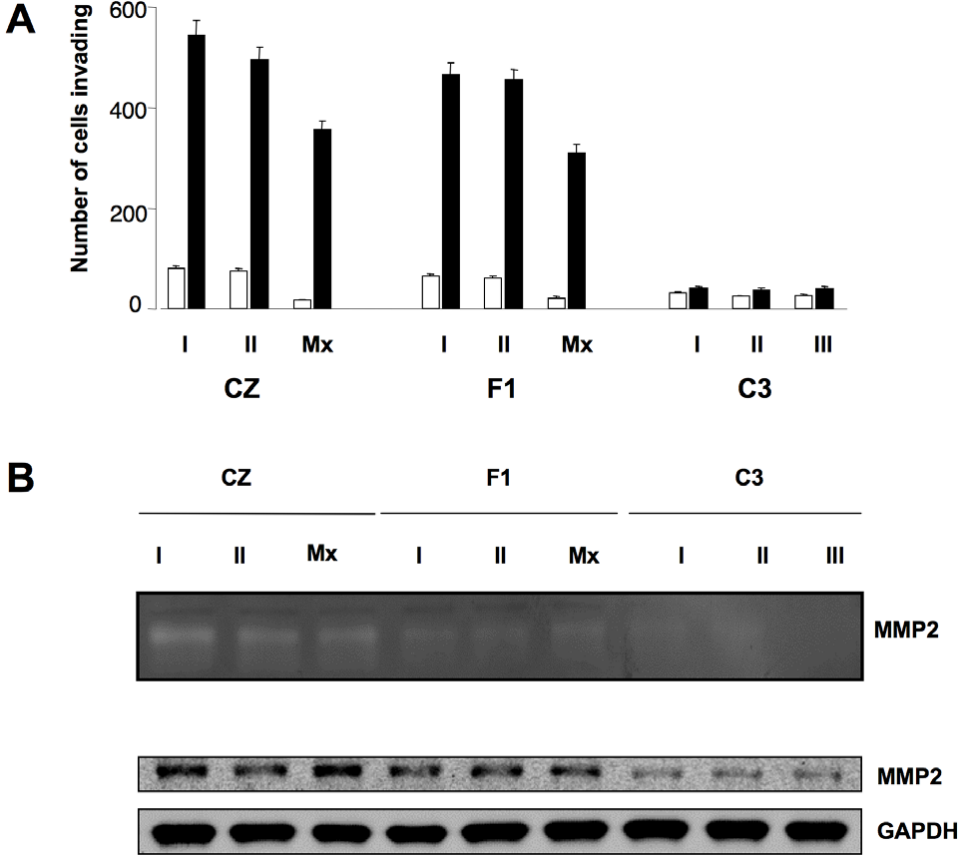
Metastatic tumor cells invade Matrigel via an MMP-2-mediated pathway. A) Migration assay was carried out as in Figure 2A except the upper surface of the filter was coated with Matrigel. B) Upper – Zymography with gelatin as ‘in gel’ substrate and conditioned media from each of the tumor lines. Lower – Immunoblot for MMP-2 in tumor cell lysates.

An important difference between CZ and C3 osteosarcoma cell lines was seen using a ‘Matrigel’ invasion assay in Boyden chambers with coated membranes [22]. CZ osteosarcoma cells readily invaded across the extracellular matrix barrier in response to C3 bone tumor cell conditioned medium (Figure 3A). Migration of these cells through the barrier was stimulated 6–8 fold over a 4 hour period. Tumor cells from F1 mice behaved like those from CZ. In contrast, C3 tumor lines showed almost no ability to penetrate the coated membranes under the same conditions. The abilities of osteosarcoma cells to invade in this assay match their metastatic behavior *in vivo.* Tumor lines established directly from metastatic lesions in lungs of CZ and F1 mice behaved similarly to those from primary tumors with respect to their properties of migration (Figure 2A and C) and invasion (Figure 3A).

### Invasion is linked to elevated levels of the metalloproteinase MMP-2 and of NFAT as an upstream regulator

The abilities of CZ and F1 tumor cells to penetrate ‘Matrigel’ suggests the involvement of one or more matrix metalloproteinases (MMPs). A zymograph assay was used to identify MMPs and estimate their levels of secretion. Conditioned medium from each of the tumor cell lines was concentrated as sources of enzyme(s). Gelatin was used as a substrate in the ‘in gel’ assay for detection of MMP-2 or MMP-9, two metalloproteinases linked to invasion and metastasis by bone tumor cells [23]. Clearing of the gel was seen at a single position of ∼70kD corresponding in molecular weight to MMP-2 (Figure 3B, top). Clearing was strongest for the three CZ tumors and somewhat weaker for the F1 tumors. Conditioned media from the C3 cell lines produced little or no clearing of the gel.

Tumor cell extracts were tested to confirm the presence of MMP-2 and to compare levels among the lines. Extracts were separated by SDS gel electrophoresis and immunoblotted with anti-MMP-2 antibody. MMP-2 was detected in each of the cell lines but levels were higher in CZ and F1 compared to C3 tumor cells (Figure 3B, bottom). Levels of endogenous MMP-2 were estimated by densitometry. Values were normalized to loading controls and averaged for the cell lines based on host of origin. The values (arbitrary units) were 1.14±0.22 for the three C3 lines, 3.79±0.27 for the F1 lines and 5.18±1.37 for the CZ lines. The tumor cells from CZ thus expresses 4 to 5 fold higher levels of the protease than those from C3. Comparison of the zymograph with the immunoblot (Figure 3B) suggests that secreted levels of MMP-2 may be even more disproportionate (CZ > C3). This would indicate that differences in the secretory pathway or extracellular activation of the enzyme may also contribute to the ability to invade. Other MMPs not directly tested for here may also be involved.

**Figure 4:**
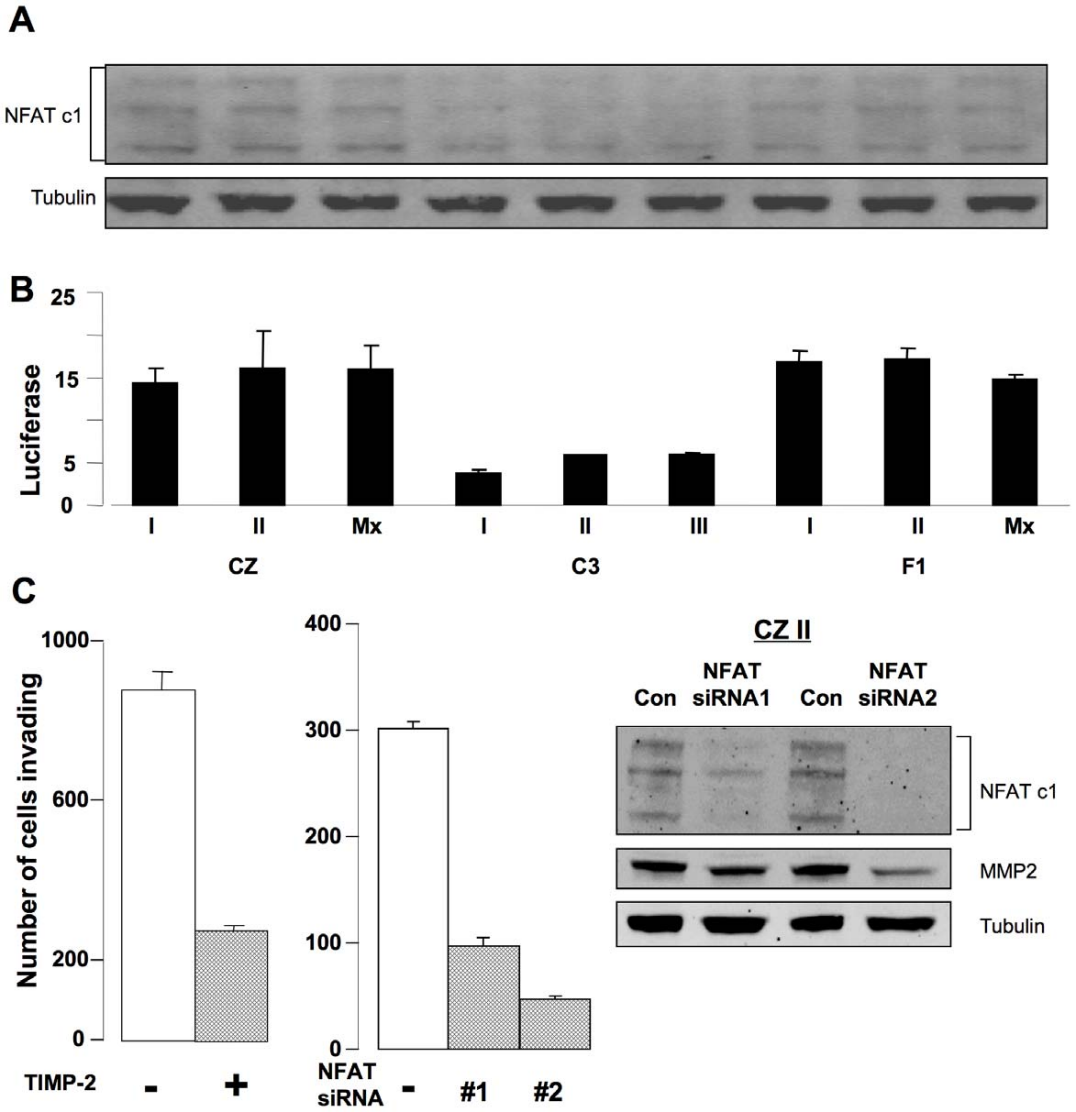
Activation of NFAT and secretion of MMP-2 are critical for invasion by metastatic bone tumor cells. A) Immunoblot for NFAT c1 in tumor cell lysates. B) Normalized luciferase activity of an NFAT reporter in tumor cells. C) Inhibition of invasion by CZ II tumor cells. Left: cells ± TIMP-2. Middle: cells ± NFAT siRNA. Right: immunoblots on cells ± NFAT siRNA.

The finding of higher levels of MMP-2 secreted by CZ and F1 compared to C3 tumor cells raises the possibility of involvement of the transcription factor NFAT [24]. NFAT is known to regulate the expression of MMPs and to be involved in tumor cell invasion [25],[26]. To test this possibility, tumor cell extracts were separated and blotted with antibody to NFATc1. This antibody detects three alternatively spliced species among which there are no well established functional differences [27],[28]. The levels of all three were elevated in tumor cells from CZ and F1 compared to C3 (Figure 4A). Relative levels of NFAT based on scanning across all isoforms were as follows (arbitrary units): for the three C3 lines 384.3±68.4, for F1 676.7±58.5 and for CZ 1038.7±157.3. To measure levels of activated NFAT, tumor cells were transfected with a luciferase reporter responsive to NFAT [29]. This reporter carries elements from the IL-2 promoter and is known to be responsive to NFAT [30]. CZ and F1 cells showed 3–4 fold activation of the reporter compared to C3 cells (Figure 4B). These results demonstrate higher levels of expression and activation NFATc1 in the metastatic compared to the non-metastatic osteosarcoma cell lines.

To test for the functional importance of MMP-2 and NFAT, ‘Matrigel’ invasion assays were carried out with the CZ II line in the presence of TIMP-2 as a specific inhibitor of MMP-2 [31],[32], or following transfection with siRNAs to suppress NFAT expression (Figure 4C). Addition of TIMP-2 resulted in a 3 to 4 fold inhibition of invasion. Two siRNAs were used to directly inhibit expression of NFAT. Both were effective in reducing NFAT levels with concomitant effects on MMP-2. Importantly, both were effective in inhibiting invasion. siRNA2 was particularly effective, reducing MMP-2 levels 4 to 5 fold and blocking invasion 6 to 7 fold. Similar results were obtained with all three CZ and all three F1 lines. Thus, treatments targeting either the protease or its upstream regulator result in inhibition of invasion. These results suggest that operation of an NFAT → MMP-2 pathway at elevated levels contributes to the metastatic behavior of osteosarcomas in CZ and F1 mice.

### The CZ mouse model of osteosarcoma differs from genetically engineered models

Several mouse models of osteosarcoma have been developed based on loss or alteration of p53 or pRb. These reflect the occurrence of osteosarcoma in families with germline mutations in these tumor suppressor genes and with their frequent somatic alterations in sporadic cases of the disease. Mice with germline loss of p53 or ones expressing certain gain-of-function mutants develop osteosarcoma along with a variety of other tumors [33]–[35]. Loss of pRb by itself has not been noted to give rise to osteosarcoma, but when coupled to p53-deficiency or mutant p53, pRb deficiency potentiates the development of the disease [36],[37]. Variable rates of metastasis are found in these models. Interestingly, the frequency of p53 loss in osteosarcoma patients with localized disease was found to be the same as in patients with metastatic disease [38], suggesting that events beyond loss of p53 are important in metastasis. The events that give rise to metastasis following disruption of the transcriptional regulatory functions of these tumor suppressors are not known.

**Figure 5:**
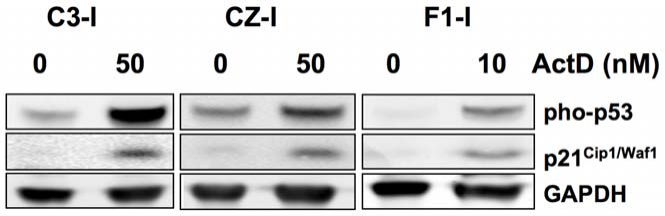
Induction of p53 and p21^Cip1/Waf1^ in a DNA damage response in metastatic and non-metastatic bone tumor cell lines. Cell extracts were separated by SDS-PAGE and immunoblotted as indicated. ActD – actinomycin D.

The predisposition to bone tumor metastasis in Py-infected CZ mice is dominantly inherited and presumed to be independent of p53 or pRb loss. Neither CZ nor C3 mice develop spontaneous tumors at a rate that would suggest the absence or altered function of p53 or pRb. Polyoma is unusual among the oncogenic DNA tumor viruses in its failure to inhibit or degrade p53 in tumors [39]. The possibility of spontaneous loss of p53 in CZ osteosarcomas was nevertheless investigated. Tumor cell lines from CZ, C3 and F1 mice were examined for p53 expression and for their response to DNA damage (Figure 5). Cells were exposed to actinomycin D (10–50 nM) for 24 hours. This led to phosphorylation on serine-18 (serine-15 in humans) and accumulation of p53 and to induction of p21^Cip1/Waf1^, as previously shown for cells in culture productively infected by the virus [40]. Similar results were found for the other lines (data not shown). pRb function is disrupted by binding to Py large T antigen [41]. However, the ability of the virus to induce tumors does not depend on this interaction [11], and metastatic mammary tumors develop without large T in middle T transgenic mice [16]. The large T-pRb interaction is expected to occur equally in tumors of CZ, C3 and F1 mice. While disruption of pRb by large T may be necessary for metastatic behavior, it is not sufficient. It thus appears that the difference between CZ and C3 mice with respect to bone tumor metastasis is based on host genetic factor(s) unrelated to p53 loss or to the action of the virus on pRb.

As in the human disease, the downstream effectors of metastasis in the engineered mouse models remain largely unknown. Elevated expression of MMP-2 and NFAT are important factors in the invasive behavior of CZ osteosarcoma cells *in vitro*. Enhanced operation of a pathway linking these factors provides a plausible mechanism contributing to the metastatic behavior of osteosarcomas in CZ mice. The genetic determinant(s) of metastasis in this system nevertheless remain unknown. Factors that impinge on calcineurin activation of NFAT [29], on secretion or activation of MMPs, expression of specific integrins involved in tumor cell invasion [42], and microenvironmental influences in the lung are among many additional factors that may be involved. Mapping and identifying genetic determinants in this system will be necessary to confirm the importance of the NFAT→MMP-2 pathway and to fully establish the molecular basis of metastasis.

## Materials and Methods

### Induction of tumors in mice

Czech II/E mice (Jackson Laboratory, Bar Harbor, ME) and C3H/BiDa mice (National Cancer Institute, Frederick, MD) were maintained in our SPF barrier animal facility. No spontaneous tumors were noted. Newborn mice were inoculated i.p. with ∼50 µl of a crude virus suspension (RA strain, 2−5×10^6^ PFU), transferred to a separate facility and monitored for tumor development as described [14]. The methods for mice use and care were approved by the Harvard Medical Area Standing Committee on Animals (“HMA IACUC”), and are in accordance with PHS policy on Care and Use of Laboratory Animals under the guidance of the Office of Laboratory Animal Welfare (OLAW) within the NIH. Histology was carried out through the Rodent Histopathology Core of the Dana Farber-Harvard Cancer Center. Screening for lung metastases was carried out by step-sectioning and microscopic examination. A total of five sections (5 µ) taken 100 µ apart were examined.

### Osteosarcoma cell lines and conditioned media

Osteosarcomas were removed at necropsy, cut into small fragments, washed and digested at 37° C overnight in medium containing 200 U/ml Collagenase, Type 1 (Worthington). Cells were spun out, resuspended and plated in DMEM containing 10% FBS. Cells were expanded initially to five 10 cm dishes and frozen. All experiments were carried out within 2–5 further passages. CZ-I and CZ-II are from osteosarcomas in two individual CZ mice. CZ-Mx is a lung metastasis from the same mouse as CZ-I. C3-I, C3-II and C3-III are non-metastatic osteosarcma lines from three individual C3 mice. F1-I and F1-II are from two F1 mice. F1-Mx is a lung metastasis from the same mouse as F1-I. Conditioned medium was prepared from confluent cultures of cells grown in DMEM with 10% FBS. Cultures were washed and incubated at 37°C overnight in serum-free DMEM. Media were concentrated ten fold using centrifugal filter devices (Millipore Corporation). Concentrates were filter sterilized and stored at −80°C. Levels of OPN and VEGF in conditioned media were determined by double sandwich ELISA.

### Assays for migration and invasion

Tumor cell migration and invasion were assayed using Boyden chambers [22] with polycarbonate membranes (8 µm pore size; 6.5 mm diameter from Corning Inc., Corning, NY). Tumor cells (3×10^5^/well in DMEM with 0.1% BSA) were placed in the upper compartment and either conditioned medium or recombinant osteopontin (4 µg/well; R&D Systems, Inc., Minneapolis, MN) in the lower compartment. Incubation was at 37°C for 1 h. Where indicated, conditioned medium was neutralized with anti-OPN or anti-VEGF antibody (5 µg/well; R&D Systems, Inc). After removal of cells from the upper side of the filter, cells on the bottom side were stained by crystal violet and counted over 7 randomly chosen fields. Invasion assays were performed in the same manner except that the polycarbonate filters were coated with ECM gel (20 µg/well; Sigma). Tumor cells (5×10^5^) were added to the upper chamber and incubated for 4 h at 37°C.

### Gelatin zymography

Procedures were essentially as described [43]. Acetone precipitates of concentrated conditioned media were resuspended and separated on 10% polyacrylamide gel containing 1 mg/mL gelatin.

### Immunoblotting

Cell extracts were separated by SDS-PAGE, transferred and blotted with antibody against NFAT c1 (Santa Cruz Biotech, Santa Cruz, CA), MMP-2 (Santa Cruz Biotech), phospho-p53 (ser-15) (Cell Signaling, Beverly, MA), or p21 (C-19) (Santa Cruz Biotech). Membranes were washed and incubated with Alexa fluor 680 anti-mouse and 800 anti-rabbit IgG antibodies (Invitrogen, Carlsbad, CA). Odyssey infrared imaging system (LI-COR Biosciences, Lincoln, NE) was used to reveal band intensities and integrated intensity values were obtained using LI-COR Odyssey software (Li-COR Biosciences).

### Luciferase assay

Cells were cotransfected with pTL-Renilla vector and pGL3 basic or pGL3 containing IL2 promoter by using Lipofectamin 2000 (Invitrogen) according to the manufacturer's protocol. At 24 hr, cells were harvested, washed and lysed with 100 µl of Passive lysis buffer (Promega, Madison, WI) at 4°C for 15 min. Twenty microliters of the cell lysate and a Dual assay kit (Promega) were used to measure fluorescence intensities. Firefly luciferase activities were normalized to those of renilla luciferase.

### siRNA transfection and TIMP-2 treatment

Cells were transfected with NFATc1 siRNA (NFATC1MSS275981 and NFATC1MSS275982; Invitrogen) or Stealth RNAi Negative Control Duplex (Invitrogen) using Oligofectamin and Opti-MEM medium (Invitrogen). Levels of NFAT at 24 hr post- transfection, were determined by immunoblotting. TIMP-2 (400 ng/ml, Sigma) was used to pretreat CZ and F1 bone tumor cells for 30 min and was added to the upper and lower chambers during the assay.

### Accession numbers (UniProtKB/Swiss-Prot)

72 kDa type IV collagenase **: P33434** (MMP2_MOUSE)

Nuclear factor of activated T-cells, cytoplasmic 1 **: O88942** (NFATc1_MOUSE)

Osteopontin **: P10923** (Osteopontin_MOUSE)

Cellular tumor antigen p53 : **P02340** (P53_MOUSE)

Metalloproteinase inhibitor 2 : **P25785** (TIMP2_MOUSE)
